# Supplementary material for: Natural Molecules in the Management of Polycystic Ovary Syndrome (PCOS): An Analytical Review
Source: Nutrients. 2021 May 15;13(5):1677. doi: 10.3390/nu13051677 (PMC8156462; doi:10.3390/nu13051677)
Supplement: Supplementary file 1 [file nutrients-13-01677-s001.zip › nutrients-1197651 - Supplementary version 3.pdf]

nutrients

Supplementary

Natural Molecules in the Management of Polycystic Ovary Syndrome (PCOS): An Analytical Review

Matteo Iervolino [1], Elisa Lepore [1], Gianpiero Forte [1], Antonio Simone Laganà [2,5], Giovanni Buzzaccarini [3] and Vittorio Unfer [4,5,*]

Table S1. Supplements and mechanisms of action. Description of the main mechanisms of action of the natural supplements mentioned in the review in relation to individual symptoms of PCOS.

**Supplements**		**Mechanisms of action on PCOS pathological features**
Inositols	Myo-inositol	Involved in the expression of glucose transporters and in cellular glucose uptake
		Decreased levels of insulin and androgens (free testosterone) and the HOMA-index
		Increased levels of sex hormone binding globulin (SHBG)
		Improved the rate of ovulation and regulated the frequency of menstrual cycles
		Improved oocyte quality and numbers mature oocyte
	D-Chiro-inositol	Involved in glycogen synthesis and storage
		High doses of D-Chiro-Ins understandably decreased oocyte quality and ovarian response
	Myo-inositol:D-Chiro-inositol (40:1 ratio)	Reduced free testosterone levels; increased sex hormone-binding globulin levels (SHBG)
		Restored normal histological features and a proper thickness ratio of theca/granulosa cell layer (TGR), suggesting that the treatment efficiently reversed the androgenic phenotype on a PCOS mouse model
		Improved levels of low-density lipoproteins (LDL), high-density lipoprotein (HDL) and triglycerides (TG), at the same time reducing fasting and circulating insulin levels
		Restored ovulation
		Improved oocyte quality and ovarian quality
		Normalized crucial parameters (progesterone, LH, SHBG, estradiol, and testosterone), also reducing the risk of cardiovascular related problems
Resveratrol		No beneficial effects on improving insulin sensitivity
		No beneficial effects on fat mass, adipocyte size, and estrus cyclicity
		Inhibited expression of pro-inflammatory cytokines
		Antioxidant and anti-apoptotic properties
		Anti-deciduogenic function in uterine endometrial tissue
		Inhibited expression of Cellular retinoic acid-binding protein 2 (CRABP2-RAR), avoiding the decidualization process and decidual senescence
		Deacetylation of crucial decidual genes encoding for Prolactin (PRL) and Insulin-like growth factor-binding protein-1 (IGFBP1)

Flavonones & Flavonoids	Naringenin	Cytoprotective and anti-inflammatory effects
		Reduced testosterone and estradiol levels in PCOS women
		Antioxidant effects by increased the concentrations of enzymes involved in ROS scavenging
		Preventing weight gain associated with PCOS
		Reduced serum glucose levels of PCOS rats
	Rutin	Ameliorating obesity and insulin resistance in obese mice, by enhancing the activity of the BAT and inducing the formation of beige adipocytes in WAT
		Significantly activation of BAT ameliorating PCOS phenotype including hyperandrogenism, cyclicity and infertility
Vitamin C		Antioxidant activities
		Important role in the regulation of the menstrual cycle and ovarian function
		Stimulates progesterone and oxytocin production
Vitamin E		Improve endometrial thickness in women with idiopathic infertility
		Negligible effects on pregnancy rate
		Antioxidant properties
		Cotreatment with coenzyme Q10 proved to increase circulating levels of SHBG
		Reduced free plasma testosterone concentrations
Vitamin D		It can support metformin effect on regulation of menstrual cycle irregularity in vitamin D-deficient/insufficient PCOS women
		No significant changes in gonadotropins or IGF-1 system
		Reduced insulin resistance and hyperandrogenism; improved lipid metabolism
		Decreased plasma glucose one-hour after the oral glucose tolerance test (OGTT)
		Greater thickness of the endometrium and a better chance of getting pregnant
		Enhanced androgen synthesis and improved abnormal folliculogenesis (attenuated effects of AGEs in women with PCOS)
		Influences the expression of genes codifying for receptors of AGEs (RAGEs), downregulating RAGEs mRNA and protein expression

Omega-3	Reduced proinflammatory cytokines, due to decreased high-sensitivity C-reactive protein (hs-CRP) and increased adiponectin levels
	No indications that omega-3 fatty acids directly affect BMI, fasting insulin, fasting glucose, and levels of HDL, FSH, LH, SHGB and total testosterone
	Related side effects: mild gastrointestinal discomforts, intestinal gas, nausea, diarrhea and headache
	Contra-indicated during antiplatelet and anticoagulant treatment
	Avoiding EPA administration during pregnancy because of the possible competitive effects with arachidonic acid, which is essential for growth processes at the foetal stage
